# Repurposing phenothiazines for cancer therapy: compromising membrane integrity in cancer cells

**DOI:** 10.3389/fonc.2023.1320621

**Published:** 2023-11-23

**Authors:** Syrina Fred Mehrabi, Sabina Elmi, Jesper Nylandsted

**Affiliations:** ^1^ Danish Cancer Institute, Membrane Integrity, Copenhagen, Denmark; ^2^ Department of Molecular Medicine, University of Southern Denmark, Odense, Denmark

**Keywords:** phenothiazines, repurposing, annexins, membrane biophysical properties, membrane integrity, cancer treatment, membrane repair, plasma membrane

## Abstract

The limitations of current cancer therapies, including the increasing prevalence of multidrug resistance, underscore the urgency for more effective treatments. One promising avenue lies in the repurposing of existing drugs. This review explores the impact of phenothiazines, primarily used as antipsychotic agents, on key mechanisms driving tumor growth and metastasis. The cationic and amphiphilic nature of phenothiazines allows interaction with the lipid bilayer of cellular membranes, resulting in alterations in lipid composition, modulation of calcium channels, fluidity, thinning, and integrity of the plasma membrane. This is especially significant in the setting of increased metabolic activity, a higher proliferative rate, and the invasiveness of cancer cells, which often rely on plasma membrane repair. Therefore, properties of phenothiazines such as compromising plasma membrane integrity and repair, disturbing calcium regulation, inducing cytosolic K-RAS accumulation, and sphingomyelin accumulation in the plasma membrane might counteract multidrug resistance by sensitizing cancer cells to membrane damage and chemotherapy. This review outlines a comprehensive overview of the mechanisms driving the anticancer activities of phenothiazines derivates such as trifluoperazine, prochlorperazine, chlorpromazine, promethazine, thioridazine, and fluphenazine. The repurposing potential of phenothiazines paves the way for novel approaches to improve future cancer treatment.

## 1 Introduction

Cancer remains a complex and heterogeneous disease that poses a significant global health challenge. Drug resistance and side effects restrict the effectiveness of existing therapies, emphasizing the need for new and effective treatments (1). In recent years, drug repurposing has emerged as a promising strategy for identifying new anticancer agents, given its potential to rapidly develop drugs with established safety profiles and known pharmacokinetic properties (2).

Phenothiazines belong to important antipsychotic drugs used for schizophrenia and bipolar disorder treatment (3, 4). They demonstrate a broad spectrum of biological activities in mammalian cancer cells, as well as pathogenic bacteria and fungi with antipsychotic, antiemetic, antihistaminic, and anti-inflammatory properties (5, 6). Beyond psychiatric use, phenothiazines may act as potential anticancer agents, targeting processes involved in tumor growth and metastasis (7, 8).

Cancer cells are exposed to membrane stress due to their enhanced metabolic activity (9), making them more reliant on an effective plasma membrane repair mechanism to restore membrane integrity and avoid cell death (10). Annexins, a group of essential plasma membrane repair proteins, are often overexpressed in cancer cells (11, 12). They are characterized by their calcium-dependent binding to anionic phospholipids and the ability to aggregate vesicles and fuse membranes (13, 14). Despite excessive research on annexin-mediated membrane repair and annexins’ ability to accumulate and fuse with membranes (15–17), pharmacological approaches to impair membrane repair in cancer cells need to be elucidated. Compromising plasma membrane repair makes cancer cells more susceptible to membrane damage and cell death (18, 19).

Phenothiazine derivatives interfere with plasma membrane junctions, induce lipid phase separation (20, 21), and, as amphiphilic drugs, modify cell membrane properties. They achieve this by altering lipid composition, disrupting lipid rafts (22), thinning the plasma membrane (23), and modulating calcium channels (24). These properties are important aspects in cancer therapy, as phenothiazines have been shown to counteract multidrug resistance in various types of cancer cells and sensitize them to chemotherapy (25).

This review aims to provide a comprehensive overview of the molecular mechanisms underlying the anticancer activity of phenothiazines by influencing the biophysical properties of the plasma membrane. We will summarize current advances in understanding the therapeutic potential of established phenothiazines and their effects on plasma membrane integrity, while discussing the prospects of repurposing these drugs for cancer therapy.

## 2 Phenothiazines: from antipsychotics to anticancer agents

### 2.1 Structure and mechanism of actions

**Figure 1:**
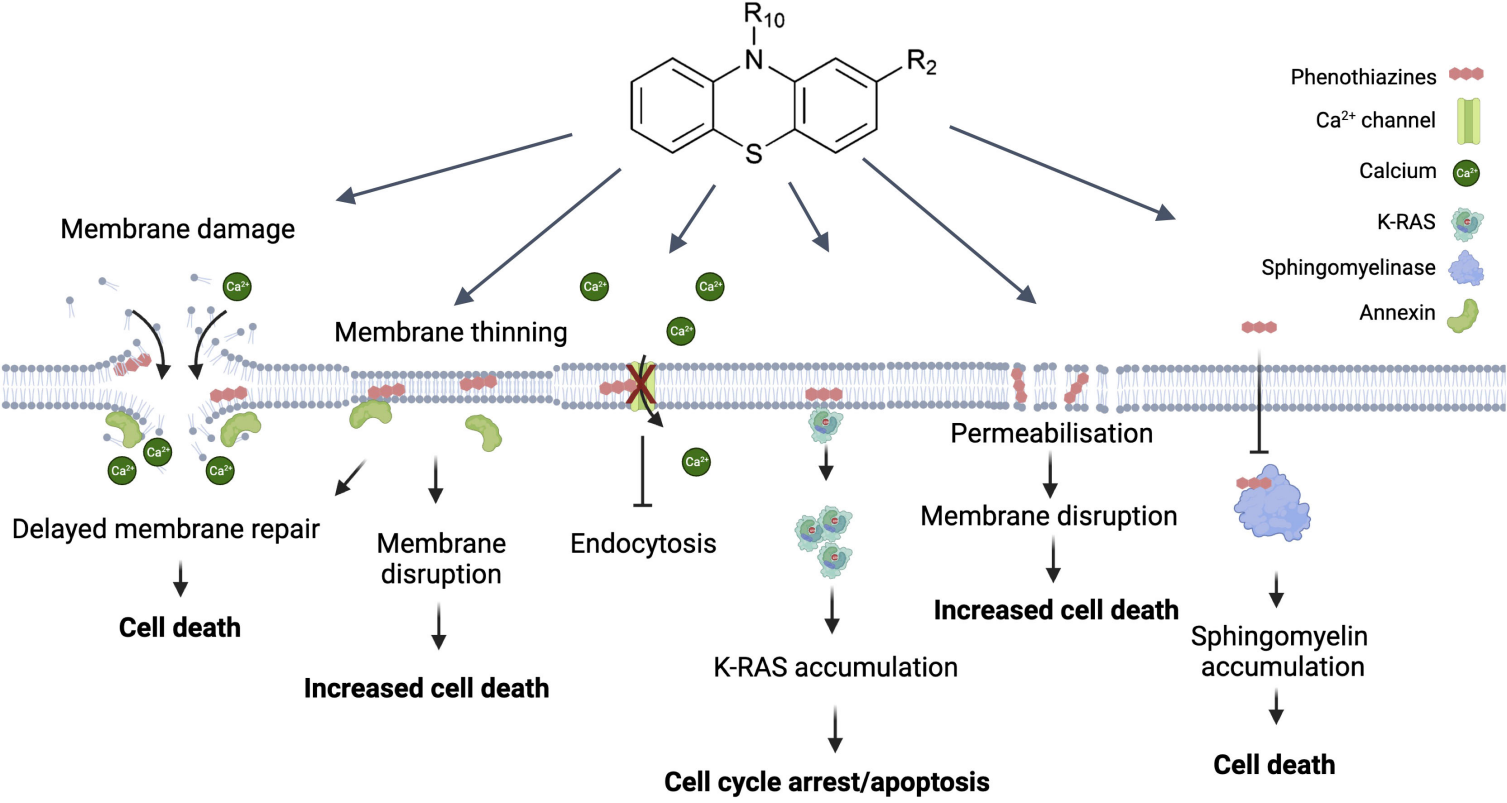
Direct effects of phenothiazine derivates on the plasma membrane and their potential as anti-cancer drugs. The most direct effect caused by phenothiazines is disruption of the plasma membrane. This causes a rapid influx of Ca^2+^ which causes depolarization of the actin filaments and activates the membrane repair machinery. The presence of phenothiazines can delay membrane resealing and thus lead to cell death. Similarly, phenothiazines can primarily induce membrane thinning and increased membrane permeabilization which can then also lead to membrane disruption. In contrast to the increased Ca^2+^ influx associated with membrane disruption phenothiazines can also inactivate the Ca^2+^ channels causing Ca^2+^ dysregulation affecting multiple cellular functions including growth. A more specific effect on growth is the interaction between phenothiazine and K-RAS, causing K-RAS to dissociate from the plasma membrane and accumulate in the cytosol, leading to cell cycle arrest and/or apoptosis. Finally, phenothiazines can inhibit the sphingomyelinase leading to sphingomyelin accumulation in the plasma membrane and subsequent cell death. Created with Biorender.

Phenothiazines represent a class of cationic and amphiphilic compounds characterized by the presence of two phenyl rings and thiazine ring containing sulfur and nitrogen atoms (**Figure 1**). An alkyl bridge is linked to the nitrogen atom within the thiazine ring (26). Phenothiazines are a group of heterocyclic neuroleptic agents known as dopamine receptor blockers that also affect GABA-mediated inhibitory synaptic transmission in cultured hippocampal neurons (27). Additionally, they demonstrated the capacity to inhibit voltage-gated Kv1.3 channels in T lymphocytes (28).

Phenothiazines demonstrate a broad spectrum of biological activities in mammalian cancer cells, as well as pathogenic microorganisms, which include bacteria (29), fungi, and protozoa. These compounds exhibit antipsychotic, antiemetic, antihistaminic, and anti-inflammatory properties and have been used in the treatment of a wide range of diseases (6).

Phenothiazines exert their anticancer effects through multiple mechanisms (30). They inhibit cell proliferation by targeting different stages of the cell cycle (8), including DNA repair (31) and microtubule dynamics (32). In addition, they also modulate signaling pathways such as, PDK1/Akt and MAPK/ERK1/2, which are involved in cancer progression and survival (7, 33). Other studies support the induction of apoptosis by phenothiazines through inhibiting the Akt/mTOR pathway, leading to decreased cell proliferation (33, 34).

Phenothiazines also inhibit angiogenesis, the formation of new blood vessels necessary for tumor growth, by inhibiting the production of VEGF (vascular endothelial growth factor) and VEGF-mediated signaling. Additionally, phenothiazines modulate other molecular pathways involved in angiogenesis, such as the MAPK signaling pathway (6, 35).

Furthermore, phenothiazines can induce oxidative stress by generating reactive oxygen species (ROS) or inhibiting antioxidant enzymes. This oxidative stress leads to DNA damage, mitochondrial dysfunction, and cell death. Cancer cells, which often have higher levels of oxidative stress, are particularly susceptible to this cytotoxicity (36).

### 2.2 Disruption of membrane integrity

Phenothiazines disrupt the integrity of cell membranes via their intercalation with the lipid bilayer (22). These compounds accumulate selectively within the lipid membrane and have profound effects on its biophysical properties (20, 37). By influencing membrane fluidity and organization, phenothiazines can impact crucial membrane-dependent processes such as signal transduction, ion channel activity, and membrane repair mechanisms (23). The study of the complex interaction between phenothiazines and membrane dynamics gives significant insight into their multiple pharmacological activities and highlights their potential as therapeutic agents in various contexts of disease.

## 3 Plasma membrane integrity is essential for cell life

### 3.1 Membrane integrity in maintaining cellular homeostasis

The plasma membrane is a vital component of all living cells, serving as a selective barrier between the intracellular and extracellular environments. Maintaining membrane integrity is essential for cellular homeostasis, since membrane disturbances may impair function and result in cell death (38, 39). The plasma membrane is composed of a phospholipid bilayer containing various proteins and molecules. Integral membrane proteins, like receptors, transporters, and channels, are pivotal for specific cellular functions and external interactions. The plasma membrane is involved in cell signaling through surface receptors that sense external signals like hormones or neurotransmitters. These signals are conveyed into the cell, triggering specific responses crucial for communication, growth, differentiation, and survival (40).

Furthermore, the plasma membrane contributes to maintaining cellular homeostasis by regulating the balance of ions, nutrients, and waste products. This regulation ensures that the intracellular environment remains stable and suitable for cellular function. Additionally, the membrane facilitates cellular adhesion, allowing cells to interact with neighboring cells and form tissues and organs (41).

Beyond its structural and functional roles, the plasma membrane is dynamic and capable of remodeling and reorganizing in response to various stimuli (41). It can change its shape, form specialized structures such as microvilli or pseudopodia, and undergo processes such as endocytosis and exocytosis, allowing an internalization or release of substances (42).

### 3.2 Perturbations in membrane integrity associated with cancer development and progression

Compromised membrane integrity is closely associated with the induction of cell death pathways (38). Understanding repair mechanisms is crucial for unraveling the complex relationship between membrane integrity and cellular homeostasis, offering therapeutic opportunities in conditions like cancer. A notable characteristic of metastatic cancer cell membranes is that lipid content may change over time. For example, cells undergoing metastasis reduce their cholesterol levels and increase their fluidity and plasticity to facilitate penetration into blood arteries (43). Additionally, reduced cholesterol levels disrupt lipid raft formation and can affect the localization and activity of membrane-associated proteins, influencing important cellular processes such as proliferation, apoptosis, and invasion (44, 45). Calcium ions (Ca^2+^) are crucial molecules involved in intracellular signaling, which is important for cell proliferation and survival (46). Repair of plasma membrane wounds is initiated by the influx of Ca^2+^ and the recruitment of Ca^2+^-regulated proteins, particularly annexins (13, 47). Annexin protein family members (ANXA) (in mammals: ANXA1-11 and ANXA13) play a crucial role in membrane fusion and wound healing (14). They are recruited to the damaged plasma membrane by binding to negatively charged phospholipids, facilitating membrane reshaping and fusion, thus promoting effective resealing. Annexins have diverse properties that contribute to membrane shaping and enable customized responses for efficient repair (15, 16, 48, 49).

Understanding membrane repair mechanisms opens novel avenues to target these processes and develop novel potential therapeutic strategies.

## 4 Changes in the structure of the cell membrane in response to phenothiazines

Multiple studies support the notion that phenothiazines exert therapeutic effects by modulating membrane function (72, 73). Derivatives of phenothiazine have demonstrated the ability to induce a range of alterations in the structure of cell membranes through molecular interactions with lipid bilayers in cancer cells (25). In this context, we have investigated the impact of various well-known phenothiazines on the plasma membrane of cancer cells and their ability to inhibit repair upon membrane damage (**Table 1**).

**Table 1:** Phenothiazines effecting cell membrane integrity and their respective anti-tumor activities.

Phenothiazines	Anti- tumor activity	Cancer Types	Effects on Membrane Integrity	*In Vivo*/Vitro Efficacy
Chlorpromazine	Induces cytotoxic autophagy in glioblastoma cells via ER stress and the unfolded protein response, causes mitotic arrest through KSP/Eg5 inhibition (50), affects CcO, complex IV in chemo resistant cells in GBM (51).	Leukemia (52), GBM (51), EC (53)	Reduces the association of K-Ras with the plasma membrane and increases its exchange between membrane and cytoplasmic pools leading to apoptosis (54).	*In vitro*: CPZ suppresses *in vitro* wound healing of PANC-1 GFP-K-Ras (G12V) cells and inhibits colony formation in soft agar (54). *In vivo*: cell-cycle arrest at the G2/M phase in rat C6 glioma cells, selectively inhibits growth and proliferation of chemo resistant glioma cells expressing COX4-1 (51).
Fluphenazine	Inhibits sphingomyelinase and causes cellular sphingomyelin accumulation (55), targets the Akt and Wnt signaling, induces DNA alterations and affects migration (8, 36, 56).	liver (36), oral and ovarian cancer (36), LC (8, 56), TNBC (56).	Alters membrane integrity by perturbing lipid bilayer structure and affecting membrane dynamics (23). Potentially, affects membrane repair processes (36).	*In vitro*: Induced G0/G1 cell cycle arrest and mitochondria mediated intrinsic apoptosis (8). *In vivo*: induced cancer cell apoptosis in a TNBC subcutaneous xenograft mouse model (56).
Prochlorperazine	Inhibits the P2X7 receptor on plasma membrane (57), enhance the efficacy of anti-tumor mAbs (58), Blocks D2 dopamine receptors (57).	TNBC (58), LC (59) GBM (57).	Calcium channel blockade (57), disrupts the structural organization between lipids and proteins in microsomal membranes (59).	*In vitro*: PCZ exhibits a synergistic effect on cancer cell death, both *in vitro* and in xenograft models, and improves the overall survival of mice (59). *In vivo*: alters EGFR distribution, reversibly inhibit the endocytosis of membrane proteins targeted by therapeutic monoclonal antibodies (58).
Promethazine	Initiating of autophagy-associated apoptosis through AMPK activation and PI3K/AKT/mTOR inhibition (60), promotes apoptosis by suppressing the PI3K/AKT signaling pathway (61), hinders proliferation and induces autophagy by increasing LC3II and p62 levels in cancer cell lines (62).	CML (60), CRC (61), SCLC (63), PDAC (62).	Indicates an early phosphatidylserine externalization followed by later plasma membrane permeabilization (60).	*In vitro*: Exhibits potent and specific cytotoxicity against various leukemia cell types through the activation of AMPK and the inhibition of the PI3K/AKT/mTOR pathway (60), impedes cell proliferation and triggers autophagy by elevating the levels of LC3II and p62 in human pancreatic ductal adenocarcinoma (PDAC) cell lines (62). *In vivo*: Reduces the growth of both mouse and human SCLC by inducing cell death (63).
Thioridazine	Induces eryptosis (64), targeting and inhibiting the PI3K/Akt/mTOR/p70S6K signaling pathway, leading to cell cycle arrest, apoptosis, and cytotoxic effects (65, 66), modulates endothelial cells and impedes angiogenesis via the VEGFR-2/PI3K/mTOR pathway, triggers autophagy by upregulating AMPK activity (67).	TNBC (65), cervical and endometrial cancer (34), OC (35), GBM (67)	Membrane permeabilization (66); triggering of cell membrane scrambling with increase of phosphatidylserine abundance at the cell surface, Thioridazine is partially effective by activation of p38 kinase and by increase of cytosolic Ca^2+^ concentration (64).	*In vitro*: induces autophagy in glioblastoma multiforme (GBM) cell lines and upregulates AMPK activity (67), inhibited the viability and migration of TNBC cells (65). *In vivo*: Strong antiproliferative effects on B16 melanoma cells, inducing DNA fragmentation and increasing the expression of Caspase-3, a key mediator of apoptosis (68), TZ reduces growth and angiogenesis in ovarian cancer by reducing the phosphorylation of VEGFR-2 and inhibiting PI3K/mTOR signaling in xenografts (35).
Trifluoperazine	Disrupts ANXA-mediated plasma membrane repair (23), induces G0/G1 cell cycle arrest and inhibit proliferation and apoptosis of tumor cells (69), suppress tumor cell growth (70, 71).	Metastatic melanoma (69), TNBC (70), GBM (71)	disrupts ANXA-mediated plasma membrane repair (23), reduces plasma membrane fluidity by intercalating into the lipid bilayer, thins the membrane bilayer and making it more fragile (23, 69).	*In vitro*: Induced G0/G1 cell cycle arrest via decreasing the expression of both cyclinD1/CDK4 and cyclin E/CDK2 in TNBC (70), decreased cell viability and proliferation, colony formation and spheroid growth on metastatic melanoma (69). *In vivo*: Increased the radiosensitivity of GBM, resulting in increased tumor cell death and prolonged animal survival (71), cytotoxic effects on melanoma brain metastases (69)

### 4.1 Trifluoperazine (TFP)

TFP has been shown to induce lysosomal membrane permeabilization (69) and conformational alterations in membrane organization, caused by a reorganization of the surrounding lipids (74).

Moreover, TFP offers great potential as an inhibitor of plasma membrane repair that sensitizes cancer cells to plasma membrane damage (23). The findings of our study demonstrate that TFP intercalation in the plasma membrane induces membrane thinning and sensitizes cells to membrane injury and cell death. Moreover, the cationic properties of TFP compromise ANXA2 binding to the membrane, delaying the recruitment of ANXA proteins and weakening their attachment to the membrane. This further reduces their ability to induce ANXA4 and ANXA6-mediated membrane curvature around the damaged areas of the membrane (49, 75). This cascade of events initiated by TFP compromises the overall membrane repair response, leaving ruptures unrepaired and sensitizing cells to potential spontaneous injury and death (23).

Other *in vitro* experiments have shown that TFP induces cell cycle arrest and apoptosis in different cancer cell lines, including triple-negative breast cancer (TNBC) and brain metastases (70). Both *in vitro* and *in vivo* xenograft models demonstrated TFP binding to calmodulin (CaM), inhibiting glioblastoma proliferation and invasion by targeting Ca^2+^ signals (76). This interaction may have a significant impact on the inositol 1,4,5-triphosphate receptor (IP3R), a Ca^2+^ release channel located in intracellular Ca^2+^ stores, and IP3R-mediated Ca^2+^ release (24, 77, 78). Moreover, TFP has demonstrated the ability to enhance the radiosensitivity of glioblastoma multiforme (GBM), resulting in increased tumor cell mortality and extended survival (71). These findings highlight the potential of TFP as an anticancer agent with the ability to sensitize cancer cells to plasma membrane damage and target Ca^2+^ signals in glioblastoma, offering new possibilities for therapeutic interventions in cancer treatment.

### 4.2 Prochlorperazine (PCZ)

PCZ, as primarly an antipsychotic and antiemetic medication, shows promise in cancer therapy by targeting specific cancer-related molecules, including KRAS mutants. PCZ binds to KRAS mutants’ GTP-binding sites, inhibiting their continuous activation. Additionally, the combination of PCZ and irradiation treatment synergistically increases the radiosensitivity of xenografted mice by downregulating the Ras/Raf/MEK/ERK signaling pathway and reducing the clonogenic survival of KRAS-mutant NSCLC. This combination treatment activates p-ATM, p53, and p21 proteins, leading to cell cycle arrest (59). PCZ also modulates plasma membrane P2X7 receptors, leading to the inhibition of P2X7-mediated Ca^2+^ entry, and potential impacts on cellular processes such as proliferation and apoptosis (57). PCZ disrupts the structural organization between lipids and proteins in microsomal membranes, thereby altering the activity and regulation of integral membrane proteins (79). Moreover, studies have shown that PCZ can reversibly inhibit the *in vivo* endocytosis of membrane proteins (58).

### 4.3 Chlorpromazine (CPZ)

CPZ is known for its evident interactions with biological membranes. It accumulates in membranes and modulates their permeability and fluidity, contributing to the biochemical and pharmacological effects of phenothiazines (73, 80). As an antipsychotic drug, CPZ antagonizes the CNS dopamine D2 receptor (DRD2) and reduces the postsynaptic effect of dopamine (81). CPZ has also demonstrated potential as an anticancer agent through interactions with key cancer-related proteins, including p53, YAP, Ras protein, ion channels, and MAPKs, influencing cell cycle regulation, cancer growth, metastasis, resistance to chemotherapy, and stemness (50, 82). CPZ has shown a suppression of cell growth in chemoresistant glioma cells and glioma stem cells. In terms of its mechanism of action, CPZ inhibited the activity of cytochrome c oxidase (CcO, complex IV) in chemoresistant cells while leaving chemosensitive cells unaffected, and it had no impact on other mitochondrial complexes (51). CPZ also disrupts Ca^2+^ signaling, raising intracellular Ca^2+^, altering Ca^2+^ homeostasis, and causing cytotoxicity in glioblastoma cells (83, 84). Furthermore, CPZ induces endoplasmic reticulum (ER) stress and unfolded protein response (UPR), influencing cell fate through autophagy (50). The interaction between CPZ and negatively charged phospholipids has demonstrated a reduction of the link between oncogenic K-Ras and the plasma membrane, hence causing an increase in the cytosolic pool of K-Ras, followed by cell cycle arrest and apoptosis in cancer cells (53, 54).

### 4.4 Promethazine (PMTZ)

PMTZ, as an initial-generation antihistamine, antipsychotic, sedative, and antiemetic drug, has shown a wide range of effects on several cancer types. PMTZ induces cell death in leukemia by activating AMPK and inhibiting the PI3K/AKT/mTOR pathway, leading to autophagy-associated apoptosis (60, 61). In chronic myeloid leukemia (CML), increasing concentrations of PMTZ have been associated with early phosphatidylserine externalization, followed by subsequent plasma membrane permeabilization (60). In colorectal cancer (CRC), PMTZ not only suppresses the proliferation of cancer cells but also initiates mitochondrial apoptosis through the PI3K/AKT pathway (61). Additionally, research has illuminated PMTZ’s capacity to induce autophagy in pancreatic ductal adenocarcinoma (PDAC), where it functions as an antagonist of proliferation (62). Furthermore, PMTZ has demonstrated a potent inhibitory impact on the proliferation of both human and murine small cell lung cancer (SCLC). Its ability to inhibit the growth of human H82 SCLC xenografts demonstrates its potential as a diverse and effective anticancer treatment (63).

### 4.5 Thioridazine (TZ)

TZ shows promise as a multifaceted anticancer agent with the ability to induce apoptosis, inhibit tumor growth, modulate angiogenesis, and target key signaling pathways involved in cancer progression. Earlier studies have demonstrated that TZ triggers eryptosis, the programmed death of red blood cells. This process is marked by disruption of the cell membrane, resulting in heightened binding of Annexin V to red blood cells situated on the cell surface, along with an elevation in cytosolic Ca^2+^ concentration and the activating p38 kinase (64). TZ exhibited inhibitory effects on TNBC cells, both *in vitro* and *in vivo*, by targeting the PI3K/AKT signaling pathway, resulting in G0/G1 cell cycle arrest, apoptosis, and mitochondrial dysfunction. This led to tumor growth suppression and the prevention of lung metastasis in TNBC models (65). TZ possesses the capability to suppress the PI3K/Akt/mTOR/p70S6K signaling pathway and exhibits cytotoxic effects on cervical and endometrial cancer cells through the induction of cell cycle arrest and apoptosis (34, 66). Moreover, TZ was found to disrupt signaling pathways downstream of PI3K, including Akt, PDK1, and mTOR, in ovarian tumor progression via vascular endothelial growth factor receptor 2 (VEGFR-2). This suggests that TZ can modulate endothelial cell function and inhibit angiogenesis through the VEGFR-2/PI3K/mTOR pathway, making it a potential anti-angiogenic agent in ovarian cancer (OC) treatment (35). Furthermore, TZ induces autophagy in GBM cell lines and upregulates AMPK activity (67). TZ has shown a strong antiproliferative effect on melanoma by inducing DNA fragmentation and increasing the expression of caspase-3 (68). These findings highlight the potential of TZ as a therapeutic agent against cancer.

### 4.6 Fluphenazine

Fluphenazine shows promising potential as a repurposed drug for cancer treatment, effectively reducing the viability of various types of cancers such as lung, TNBC, colon, liver, brain, leukemia, oral, ovarian, and skin (36). Fluphenazine shows anticancer properties, and its antitumor activity is mainly mediated by an effect on the cell cycle, proliferation, or apoptosis. This effect is partly mediated by the inhibition of the lysosomal enzyme sphingomyelinase which leads to increased cellular levels of sphingomyelin (55). It should also be noted that this mechanism differs from other known lysosomal-disrupting agents (85, 86). Furthermore, fluphenazine’s interaction with dipalmitoyl phosphatidylcholine (DPPC) bilayers, the main component of pulmonary surfactants, leads to the disruption of the lipid bilayer and the formation of an isotropic phase at higher concentrations. These interactions contribute to its multidrug-resistant (MDR) activity, which offers a potential strategy for cancer chemoprevention (87). In the context of TNBC and brain metastases, fluphenazine hydrochloride (Flu) was investigated. Flu effectively inhibited the survival of metastatic TNBC cells, inducing arrest of the G0/G1 cell cycle and mitochondrial-mediated intrinsic apoptosis *in vitro*. Pharmacokinetic studies in mice demonstrated favorable brain bioavailability of Flu for at least 24 hours. In particular, Flu exhibited strong antimetastatic effects in a mouse model of brain metastasis, achieving an impressive 85% inhibition rate. Furthermore, Flu showed a significant inhibition of spontaneous lung metastasis without severe side effects (56). These promising findings urge further research to evaluate Flu’s potential as a treatment option for metastatic TNBC and address the urgent need for novel therapeutic approaches.

## 5 Conclusions and prospects

Repurposing drugs offers innovative solutions that can exceed standard cancer treatments in effectiveness and safety. Phenothiazines show promise against drug resistance and cancer due to their unique properties, including hydrophobicity and specific structure (2, 6, 26). They exhibit diverse effects on cancer cells, including inhibiting proliferation, disrupting cell cycles, preventing metastasis, inducing apoptosis, and enhancing chemotherapy sensitivity (61, 65, 70, 82).

Maintaining cell membrane integrity is vital for survival. Cancer cells, much like normal cells, reprogram themselves to repair damaged membranes and avoid apoptosis (38). Phenothiazines are gaining scientific attention for their impact on membrane dynamics. They interact with the lipid bilayer and profoundly disturb the biophysical properties of cell membranes, such as fluidity and lipid organization, affecting downstream signal transduction and ion channel activity (20, 37). These compounds also inhibit annexin-mediated plasma membrane repair, which induces membrane thinning and reduces annexin-mediated membrane curvature (23). Disturbances in membrane repair machinery sensitize cells to membrane ruptures, ultimately triggering a cascade of cellular responses that culminate in cell death (15, 23, 39, 49). In addition, they may influence Ca^2+^ regulation by modifying the activation of Ca^2+^ receptors such as PMCA and IP3R, hence influencing downstream signaling cascades (24, 83, 84). Furthermore, phenothiazines suppress the PI3K/AKT (7, 34, 61, 65) pathway and interfere with critical cancer-related proteins like K-RAS (54), directing cellular outcomes toward cycle arrest, apoptosis, and reduced proliferation and survival. Their involvement in disturbing membrane permeability and sphingomyelin accumulation provides insights into the complex mechanisms driving cytotoxicity (21, 55, 86, 87).

The anticancer properties of phenothiazines may vary depending on their dosage, since it has been shown that clinically significant levels (~ 1-2 µM) might promote tumor growth (88, 89). However, the membrane-compromising actions of phenothiazines seem to need greater concentrations (~ 7-15 µM) (23). Consequently, the use of higher dosages may elevate the risk of potential side effects, particularly when taken in combination with chemotherapeutic agents. The inconsistent findings regarding these antipsychotic drugs in cancer cells underscore their concentration-dependent characteristics. The role of phenothiazines in cancer treatment may not only vary in relation to concentration but also in accordance with the cancer type. Hence, it is important to evaluate both aspects, when assessing the therapeutic potential of phenothiazines.

In summary, the multifaceted effects of phenothiazines on cellular membranes present significant potential for their repurposing in cancer therapy. Their ability to disrupt membrane integrity, inhibit repair processes, and modify critical cellular pathways positions them as intriguing options for the targeted therapy of cancer. A comprehensive understanding of their interaction with membrane dynamics introduces a fresh perspective for developing innovative therapeutic approaches to combat cancer and address various pathological conditions.

## Author contributions

SM: Conceptualization, Writing – original draft, Writing – review & editing. SE: Writing – review & editing. JN: Conceptualization, Writing – original draft, Writing – review & editing.
